# Vitamin D treatment for chronic urticaria: a case report

**DOI:** 10.1186/s13256-019-2121-9

**Published:** 2019-06-25

**Authors:** Irene Yuan, Priya Katari, Marcus Shaker

**Affiliations:** 1 Geisel School of Medicine at Dartmouth, Hanover, NH USA; 2 Department of Internal Medicine, Dartmouth–Hitchcock Medical Center, Lebanon, NH USA; 3 Dartmouth–Hitchcock Medical Center, Section of Allergy and Clinical Immunology, Lebanon, NH 03756 USA

**Keywords:** Chronic urticaria, Vitamin D, Refractory, Treatment

## Abstract

**Introduction:**

Chronic urticaria is characterized by recurrent hives, with or without angioedema, persisting for 6 weeks or longer. Although often suspected by patients, in fact specific allergen triggers are infrequent. In contrast, the condition may be associated with autoimmune and thyroid disorders. While some evidence suggests an association of chronic urticaria with vitamin D levels, measurement of vitamin D or supplementation is not a part of diagnostic or treatment recommendations.

**Case presentation:**

We present a case of a 14-year-old white boy with refractory chronic urticaria who experienced prompt remission with high-dose vitamin D repletion when vitamin D deficiency was identified as an incidental finding.

**Conclusions:**

In some patients, vitamin D may have a role in the pathophysiology and treatment of chronic urticaria; however, the cost-effectiveness of routine laboratory screening in chronic urticaria is unknown.

## Introduction

Chronic urticaria affects approximately 1% of the population and is often a spontaneous phenomenon without a clearly identified allergen trigger. Treatments involve non-sedating antihistamines in escalating off-label doses, omalizumab, anti-inflammatory medications such as hydroxychloroquine, and, in some patients, immunosuppressants such as cyclosporine. We report a case of a 14-year-old white boy with refractory chronic autoimmune vitamin D-responsive urticaria.

## Case presentation

**Fig. 1:**
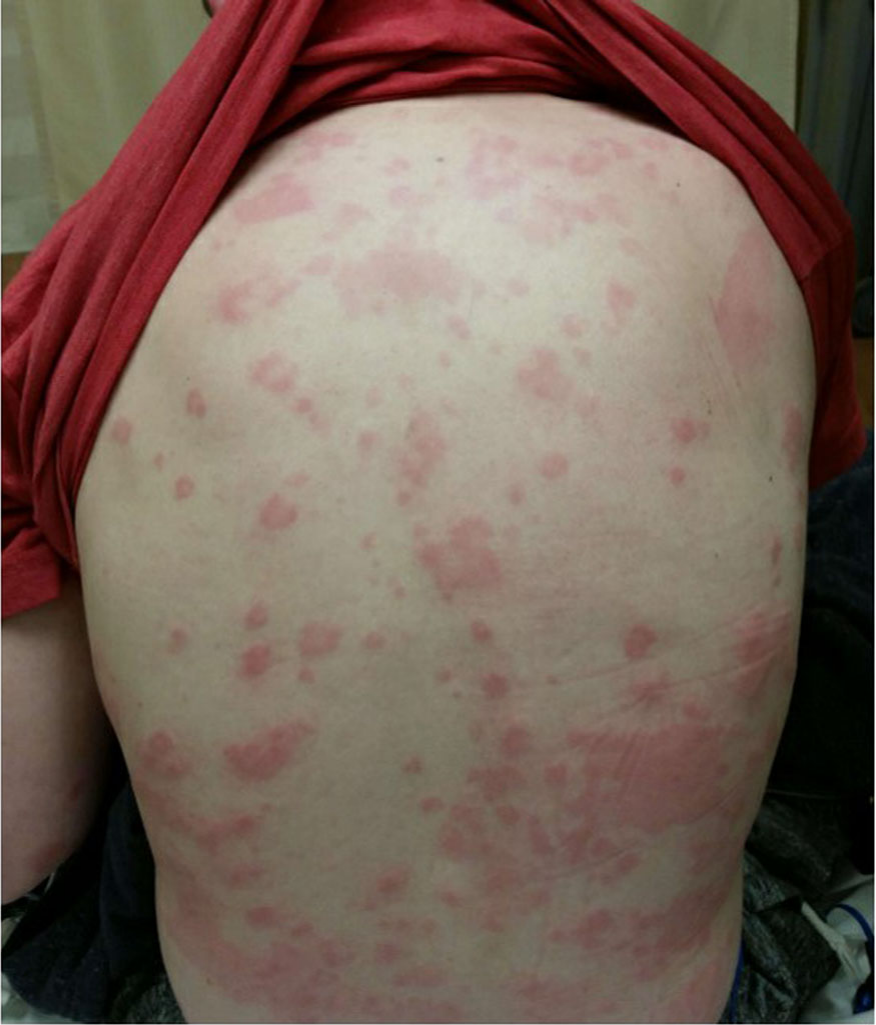
Diffuse urticaria in patient

In October 2017, our 14-year-old white male patient developed evanescent, pruritic, and bothersome urticaria that persisted over several months despite guideline-based therapy with non-sedating H_1_ blockade at four times the approved Food and Drug Administration (FDA) dosing (loratadine 10 mg twice daily and cetirizine 10 mg twice daily), H_2_ blockade (ranitidine 150 mg twice daily), and use of a leukotriene modifier (montelukast 5 mg once daily). His urticaria was recalcitrant to conventional treatment. Allergen-specific immunoglobulin E (IgE) testing was negative to dust mites, cat, dog, roach, tree pollens, grass pollens, weeds, molds, latex, and galactose-a-1,3-galactose. Laboratory evaluation revealed an elevated serum tryptase (14.3 ng/mL; normal range ≤ 11.1 ng/mL), elevated anti-IgE receptor antibody (78% CD203c basophils), and normal thyroid-stimulating hormone (TSH). A low 25(OH)-D (vitamin D) at 23 ng/mL (normal 30–100 ng/mL) was identified as an incidental finding. A trial of omalizumab (300 mg every 4 weeks) was initiated in January 2018 but failed to provide benefit after four doses, and symptoms progressed to diffuse urticaria and facial angioedema prompting emergency evaluation for the worsening flare of urticaria and angioedema (Fig. 1). On presentation to our emergency department in May 2018, vital signs were normal and a physical examination revealed patchy facial swelling, infraorbital edema, and urticarial lesions on his extremities, abdomen, and back. There was no respiratory or cardiovascular involvement. He initially received a 3-week course of orally administered corticosteroids (he did not receive an intravenously administered glucocorticoid), but when symptoms failed to improve after 2 weeks, he was started on hydroxychloroquine 200 mg daily and vitamin D supplementation (50,000 IU weekly for five doses then 2000 IU daily) in June 2018. Within 7 days of therapy initiation, his symptoms had completely resolved. He had attended four follow-up visits to the allergy clinic since his initial presentation when his therapies were weaned following this remission. Over the following months he had minor recurrences of manageable itching and minor urticaria which did not require medication.

## Discussion

Multiple factors could have contributed to our patient’s dramatic improvement, including spontaneous remission; however, the temporal association of vitamin D is notable. The time course of improvement within 1 week argues against hydroxychloroquine, as this agent typically requires several months to achieve symptom control. Hydroxychloroquine exerts its anti-inflammatory effect by protein degradation through alkalization of intracellular vacuoles and through posttranslational protein modification in the Golgi apparatus, leading to interference with antigen processing in antigen-presenting cells [1]. The resultant downregulation of autoantigenic immune response is gradual. Omalizumab is also unlikely to explain the prompt response, as our patient had received four doses of the agent previously without any improvement, and he was, in fact, experiencing worsening of his disease. Response to omalizumab is typically more rapid, with a majority of patients responding within 3 months [2].

Prior studies have associated vitamin D deficiency with urticaria. In 2010, Thorp and colleagues described significantly reduced vitamin D levels in 25 patients with chronic urticaria compared to controls [3]. A recent meta-analysis of 1655 patients demonstrated a strong association of vitamin D deficiency with chronic – but not acute – urticaria (OR 4.46, 95% CI 2.26–8.78) [4]. In addition, a randomized double-blinded controlled trial of adult patients with chronic urticaria by Rorie *et al.* demonstrated improved Urticaria Severity Scores following 12 week supplementation with 4000 IU of vitamin D_3_ per day, regardless of baseline vitamin D status [5]. In this trial, patients receiving vitamin D reported fewer days with hives, less body surface area involvement, better sleep quality, and improvement in pruritus [5].

The exact mechanism through which vitamin D improves symptoms of chronic urticaria is unclear, although vitamin D may have immunoregulatory properties and its receptors have been identified on T cells, B cells, neutrophils, macrophages, and dendritic cells [6]. Vitamin D induces greater production of interleukin-10 (IL-10), promotes CD4+ T regulatory cells, and can slow mast cell differentiation [6, 7]. In autoimmune disorders such as multiple sclerosis and rheumatoid arthritis, lower vitamin D levels are associated with higher disease activity, and supplementation in animal studies has been shown to improve symptoms of inflammatory bowel disease, diabetes, and systemic lupus erythematosus [8]. Vitamin D may have a protective effect in allergic skin conditions as well. Hata and colleagues reported that in patients with atopic dermatitis, supplementing with 4000 IU of vitamin D_3_ per day effectively increased cathelicidin expression in skin lesions after 3 weeks [9]. Cathelicidin is an antimicrobial skin peptide that is upregulated during infection or wounding, and can provide one mechanism for vitamin D’s role in strengthening the skin barrier [9].

## Conclusion

Second-generation or third-generation H_1_ antagonists (up to four times daily) remain first-line treatment for chronic urticaria, with omalizumab and cyclosporine considerations for more difficult to treat patients. It is estimated that approximately 7–8% of patients with chronic spontaneous urticaria will be refractory to all current treatments [10]. While the role of vitamin D in chronic urticaria is intriguing, it is worth noting that routine laboratory screening in chronic urticaria has been shown to have relatively low impact in regards to outcomes. Tarbox *et al.* described 356 adult patients between 2001 and 2009 who underwent screening laboratory investigations [11]. While 17% of 1872 tests ordered were abnormal, only 0.28% of the population benefited from a change in management due to an abnormal test result [11]. Further research is needed to establish the exact mechanism vitamin D may have in chronic urticaria pathophysiology, and whether or not routine laboratory screening in chronic urticaria is a cost-effective practice.

## Data Availability

All data and materials relevant to this report are enclosed.

## Abbreviations

FDA: Food and Drug Administration
IgE: Immunoglobulin E
IL-10: Interleukin-10
TSH: Thyroid-stimulating hormone
